# Among the Colleges

**Published:** 1903-05

**Authors:** F. R. Hanson

**Affiliations:** Secretary


### AMONG THE COLLEGES.

THE regular meeting of the Alumni Association of the New York American Veterinary College was held in the college building, Thursday April 2, 1903, at 2.30 P.M. The President, Dr. Wm. Herbert Lowe, in the chair. Members present: Drs. R. W. Ellis, R. F. Meiners, J. F. Winchester, W. Horace Hoskins, W. H. Lowe, Wm. Dougherty, W. C. Miller, J. L. Robertson, F. R. Hanson, T. E. Budd, W. J. Coates, H. D. Gill, Wm. Anderson.

The minutes of the previous meeting were read and approved. It was then reported that the annual banquet was to be held, in conjunction with the Alumni Association of the American Veterinary College and the New York College of Veterinary Surgeons, at the Hotel Marlborough on that evening at 7 P.M.

In the absence of the Treasurer, Dr. Miller reported that there was a balance of $40 in the treasury.

The class of 1903 was admitted to membership to this Association, as follows: Drs. W. J. Butler, W. W. Bennett, G. E. Smith, F. Glynn, S. A. Selby, C. E. Willis, F. M. Kettner, M. Smith, C. D. Huxtable, A. Berdan, J. M. Young, F. D. Owens, H. Stark.

There was a discussion on the subject of increasing the membership to make this society a more powerful factor, and to this end upon motion the President appointed the following committee representing each school to solicit new membership: Dr. W. C. Miller, Chairman; Dr. H. D. Gill, Dr. R. W. Ellis.

The following officers were elected for the ensuing year: President, Dr. W. H. Lowe; Vice-President, Dr. H. D. Gill; Secretary, Dr. W. C. Miller.

Under the head of new business, Dr. Hoskins asked for information through the President of the workings of the College for the purpose of aiding and lending the support of the Association to it in whatever way it might be of servicc. Dr. Lowe referred to Dr. Coates not knowing the workings of the College, and Dr. Coates suggested that hereafter Dr. Munn of the Council, be invited to attend the Alumni meeting, where the workings of the Council for the benefit of the College might be learned, Dr. Munn being on the Committee for the Veterinary Department. Dr. Hoskins then put it in the form of a motion that Dr. Munn be notified at a reasonable time of such meetings, inviting him to attend, which was seconded and carried.

A discussion then arose in regard to means for the advancement of the College in regard to new buildings and better facilities for working, Dr. Hoskins then made a motion that the officers and executive committees of the Alumni Associations of the A. V. C. and N. Y. A. V. C. meet jointly some time during the winter to draft resolutions for the same, and to report to the Association at the regular meeting.

The President, W. H. Lowe, then appointed the following members to act on the Executive Committee for the following year: Dr. R. W. Ellis, Chairman; Drs. Wm. Anderson, J. W. Fink, T. E. Budd, W. H. Hoskins. Motion in order to adjourn.

F. R. Hanson,
Secretary.

